# Neuropsychological Characterization of Autosomal Recessive Intellectual Developmental Disorder 59 Associated with IMPA1 (MRT59)

**DOI:** 10.3390/brainsci13071048

**Published:** 2023-07-10

**Authors:** Andre Luiz Santos Pessoa, Andrea Amaro Quesada, Paulo Ribeiro Nóbrega, Ana Priscila Oliveira Viana, Kécia Tavares de Oliveira, Thalita Figueiredo, Silvana Santos, Fernando Kok

**Affiliations:** 1 Albert Sabin Children’s Hospital, Fortaleza 60410-794, Brazil; andrepessoa10@yahoo.com.br (A.L.S.P.); kecia.oliveira@yahoo.com.br (K.T.d.O.); 2 Faculty of Medicine, State University of Ceará (UECE), Fortaleza 60714-903, Brazil; 3 The Edson Queiroz Foundation, University of Fortaleza (UNIFOR), Fortaleza 60811-905, Brazil; anapri_viana@yahoo.com.br; 4 Hospital Universitário Walter Cantídio—UFC, Fortaleza 60430-372, Brazil; paulo_r_med@yahoo.com.br; 5 Faculty of Medicine, Centro Universitário Christus, Fortaleza 60160-230, Brazil; 6 Faculty of Medicine, Federal University of Alagoas (UFAL), Maceio 57200-000, Brazil; thalita.figueiredo87@gmail.com; 7 State University of Paraíba (UEPB), Campina Grande 58429-500, Brazil; silvanaipe@gmail.com; 8 Department of Neurology, University of São Paulo (USP), São Paulo 05508-220, Brazil; fernando.kok@mendelics.com.br

**Keywords:** *IMPA1*, intellectual disability, functional dependence, neuropsychological profile

## Abstract

Biallelic loss of function of *IMPA1* causes autosomal recessive intellectual developmental disorder 59 (MRT59, OMIM #617323). MRT59 has been reported to present with significant intellectual disability and disruptive behavior, but little is known about the neurocognitive pattern of those patients. Thus, the aims of this study were: (1) to assess the cognitive profile of these patients, and (2) to evaluate their functional dependence levels. Eighteen adults, aged 37 to 89 years, participated in this study: nine MRT59 patients, five heterozygous carriers and four non-carrier family members. All of them were from a consanguineous family living in Northeast Brazil. All *IMPA1* patients had the (c.489_493dupGGGCT) pathogenic variant in homozygosis. For cognitive assessment, the WASI battery was applied in nine MRT59 patients and compared to heterozygous carriers and non-carrier family members. Functional dependence was evaluated using the functional independence measure (FIM). Patients showed moderate to severe intellectual disability and severe functional disabilities. Heterozygous carriers did not differ from non-carriers. MRT59 patients should be followed up by health professionals in an interdisciplinary way to understand their cognitive disabilities and functional needs properly.

## 1. Introduction

*IMPA1* is responsible for the production of inositol monophosphatase (IMPA1), an enzyme involved in the final step of the biotransformation of inositol triphosphate and diacylglycerol. The inositol cycle is implicated in a wide range of physiological functions such as insulin signaling, P13K/Akt signaling, cell migration, endocytosis, cell vesicle transport, exocytosis, cell proliferation, apoptosis, neurotransmitter release, hormonal secretion, histamine release in allergic responses and maintenance of homeostasis by second messengers. Dysregulation of the inositol cycle is associated with many human diseases including cancer, diabetes and neurological diseases [1]. Additionally, the enzyme IMPA1 has been the target of pharmacological and genetic studies in neuropsychiatric diseases. Lithium, which is largely used for bipolar disorder treatment, in high concentrations inhibits the enzyme IMPA1 in a non-competitive way [2,3,4].

Figueiredo et al. (2016) [5], in a prospective study for novel genetic diseases in Northeastern Brazil, described a large inbred family with nine adults with intellectual disability associated with behavioral changes such as paranoid behavior and aggression, without dysmorphological changes [5]. In this family, homozygous variants in *IMPA1* (c.489_493dupGGGCT), consisting of a 5 bp duplication, leading to a frameshift and a premature stop codon, resulting in loss-of-function, were evidenced by whole exome sequencing, causing a disease not previously reported in the scientific literature. Segregation analysis confirmed the association of homozygosis for this *IMPA1* variant with the clinical phenotype. The condition was later recognized as autosomal recessive intellectual developmental disorder 59 (MRT59 OMIM 617323) [5]. To our knowledge, there are no reports of intellectual disability in patients with monoallelic *IMPA1* pathogenic variants. Interestingly, heterozygous carriers of monoallelic pathogenic variants in the patients’ family show no clinical signs of neurologic or psychiatric abnormalities.

A recent neurophysiological study found a relative decrease in the power of the frontal theta band as well as altered variability of the alpha band without regional specificity during the open-eyed condition in these patients. For the closed-eyes condition, there was variability of the altered dominant theta frequency in the central and parietal regions [6].

Induced pluripotent stem cells (IPSC) technology to model *IMPA1* intellectual disability was used and identified that pathogenic variants affect the ability of neural progenitor stem cells to differentiate into neurons but not into astrocytes [7]. However, the main impaired cognitive functions in MRT59 patients were still not investigated. 

Therefore, the aims of the present study were to assess the cognitive profile and the functional dependence levels of these patients.

## 2. Materials and Methods

### 2.1. Participants

**Figure 1:**
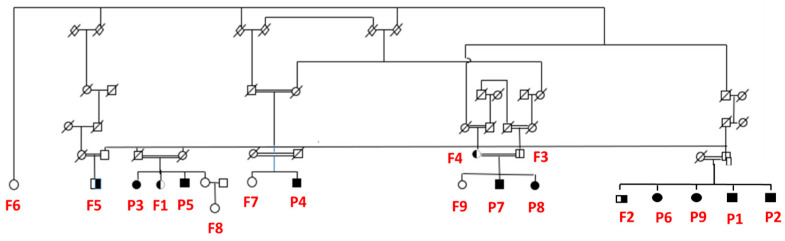
Family pedigree showing MRT59 patients, heterozygous carriers, and non-carrier family members. P1 to P9 are MRT59; from F1 to F5 are the family members who are carriers and from F6 to F9 are the non-carriers.

Eighteen adults, aged 37 to 89 years, participated in this study: 9 MRT59 patients, 5 heterozygous carriers and 4 non-carrier family members (Figure 1). All of them were from a consanguineous family living in a poor community in the hinterlands of Northeast Brazil. All *IMPA1* patients had the (c.489_493dupGGGCT) pathogenic variant in homozygosis.

To provide an appropriate age and socioeconomic status-matched control group, the 5 heterozygous carriers and the 4 non-carrier relatives of the *MRT59* individuals were used as controls. This study protocol was approved by the Ethics Committee of Hospital das Clinicas da Universidade de São Paulo (HCMUSP; CAAE: 57763116.0.0000.0065) and all participants or their legal guardians signed an informed consent, giving their permission to participate in the study.

### 2.2. Procedures

The procedures encompassed three phases: (1) acquisition of demographic and clinical variables; (2) cognitive profile assessment; and (3) functional dependence evaluation. A semi-structured interview was applied to obtain the age and socioeconomic status and clinical variables from participants. The cognitive profile was assessed by the Wechsler Abbreviated Scale of Intelligence (WASI), Brazilian version [8]. According to the DSM-V criteria for classifications of Intellectual Disability Severity, functional dependence was assessed using the Functional Independence Measure Scale (FIM). However, different from cognitive assessment, only MRT59 participants were evaluated concerning functional dependence.

We tried to perform brain magnetic resonance imaging (MRI) with spectroscopy to evaluate a possible link between structural changes, brain inositol levels and cognitive abnormalities, but due to socioeconomic reasons and difficulties in transportation and access to healthcare services in the isolated region where the patients live, we could only perform MRI scan in one patient as a proof-of-concept.

### 2.3. Sociodemographic and Clinical Variables

The socioeconomic status was computed using the educational degree as a surrogate, classified as no education or semi-illiterate; primary school (some or graduate); elementary school (some or graduate); high school (some or graduate); and college (some or graduate). Regarding clinical/genetic status, the participants were classified as MRT59, heterozygous carriers and non-carriers.

### 2.4. Wechsler Abbreviated Scale of Intelligence (WASI)

To screen the cognitive functions and the intelligence quotient (IQ) of participants, the Wechsler Abbreviated Scale of Intelligence (WASI) was applied. The WASI is used to assess people from 6 to 89 years and is composed of 4 subtests: vocabulary, block span, similarities and matrix reasoning [9].

Vocabulary assesses crystallized intelligence, word comprehension and semantic memory. Lower performance may indicate a lack of scholarly experience. Similarities evaluate crystallized intelligence, fluid verbal reasoning, auditive comprehension, verbal expression, the ability to analyze and synthesize (conceptual comparison vs. working memory), integration of knowledge, language and executive functions (cognitive flexibility). The verbal intelligence quotient (VIQ) is obtained by summing up the scores of both subtests, i.e., vocabulary and similarities. Block design measures fluid intelligence, organization, perception and visual and spatial processing, visual and spatial motor coordination, constructive praxes, visual-motor processing speed and executive processing speed (subcomponent of planning) and the ability to analyze and synthesize. Matrix reasoning also assesses fluid intelligence, abstract, analog, serial and classificatory reasoning, visual and spatial perception and executive functions (problem resolution and cognitive flexibility) [8,9,10]. The sum of block design and matrix reasoning scores gives the performance intelligence quotient (PIQ). Additionally, total intelligence quotient can be obtained by using two subtests (vocabulary and matrix reasoning; TIQ-2) or all four subtests (vocabulary, similarities, block design and matrix reasoning; TIQ-4). T scores are used for correction, which, afterward, can be transformed into scaled scores. The data shown in this study are scaled scores. IQ is classified as extremely low (69 and below), borderline (70–79), low average (80–89), average (90–109), high average (109–119), superior (120–129) and very superior (130 and above) [10].

### 2.5. Functional Independence Measure (FIM)

The FIM scale is a tool for assessing the burden of care demanded by a person to perform a series of motor and cognitive tasks of daily living. It was developed by the American Academy of Physical Medicine and Rehabilitation in 1986 for evaluating adults [11]. It is a quantitative scale, which is composed of 18 items [12]. Those items evaluate the following domains: self-care, sphincter control, mobility, locomotion, communication and social cognition (memory, social interaction and problem-solving). Each item is rated on a 7-point scale, from 1 (total dependence) to 7 (complete independence). The range of total scores is from 18 to 126. The Brazilian version of FIM was chosen due to its high reliability [13]. 

## 3. Results

### 3.1. Description of Sample

The sample was composed of 10 MRT59 patients, five carriers and four non-carriers. All MRT59 participants had no motor deficits, microcephaly or epilepsy. All were unschooled. Of the carriers, two of them were semi-literate, two of them had a college degree and one had incomplete elementary school (see Table 1). In the non-carriers, two of them were semi-literate, one had a high school degree and the other one had a college degree. Using Pearson’s chi-square test, the three groups did not differ in education (*p* = 0.078). The analysis of variance ANOVA revealed no significant differences among the three groups (F2/17 = 2.70, *p* = 0.99).

For social reasons and difficulty in transportation and access to health services, we could only perform MRI on one patient as a proof-of-concept. In this patient, brain magnetic resonance with spectroscopy did not show structural or metabolic abnormalities, including in inositol brain content.

### 3.2. Cognitive Profile

The Shapiro–Wilk test for normality showed that the distribution of the verbal intelligence quotient (VIQ), performance intelligence quotient (PIQ) and full-scale intelligence quotient using all four subtests (FSIQ-4) and using only two subtests (FSIQ-2) deviated from normality. Therefore, the Kruskal–Wallis test for non-parametric data was used for statistical analysis. Significant differences were observed among the three groups on FSIQ-4, FSIQ-2, VIQ, and PIQ: FSIQ-4 (H(2) = 13.33; *p* < 0.001), FSIQ-2 ((H(2) = 13.69; *p* < 0.001), VIQ (H(2) = 13.33; *p* < 0.001), and PIQ (H(2) = 13.33; *p* < 0.001) (see Table 2). Comparing the MRT59 group with the carriers group, the patients showed lower performance on VIQ. When comparing the MRT59 group with the non-carriers group, the first group also showed lower performance on VIQ. Differences in performance were not observed between the carriers group and the non-carriers group (*p* > 0.05).

As depicted in Table S1, all MRT59 participants showed moderate intellectual disability. They had impaired crystallized (vocabulary and similarities) and fluid intelligence (block design and matrix reasoning). Additionally, they showed impairments in verbal expression, executive functions, the ability to analyze and synthesize, fluid verbal reasoning (vocabulary and similarities) and integration of knowledge (similarities). Their PIQs (block design and matrix reasoning) indexes were low, revealing difficulties in visual and spatial perception, cognitive flexibility, executive processing speed and problem resolution. The higher FSIQ-4 was 44 in two patients. These patients also showed a higher VIQ index (52 and 51).

### 3.3. Functional Dependence

On the FIM scale, the scores of MRT59 participants ranged from 29 (participants demand assistance in 50% of tasks) to 101 points (participants demand assistance in 25% of tasks). Analyzing only the cognitive domain, scores ranged from 1.8 (total assistance) to 4.6 (minimal assistance). Individual FIM data are illustrated in Table S2.

**Figure 2:**
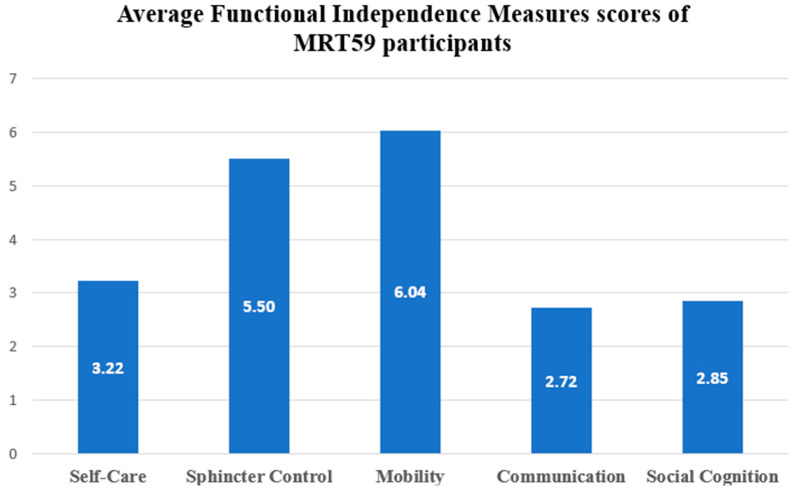
Average functional independence measure scores of MRT59 participants.

Regarding each domain, the Friedman non-parametric test revealed higher impairments in communication (C; m = 2.72 ± 1.03) and social cognition (SC; m = 2.85 ± 1.20) (*p* = 0.013). The less impaired domain was mobility (m = 6.04 ± 1.83) (see Figure 2).

Additionally, the full-scale intelligence quotient using all four subtests (FSIQ-4) scores correlated positively with two indexes of the FIM scale: communication (r = 0.79; *p* = 0.011) and social cognition (r = 0.82; *p* = 0.006). Together, low indexes on communication, social cognition and low IQ indicate, overall, a moderate to severe intellectual disability.

## 4. Discussion

The aim of the current study was to evaluate the cognitive profile and functional dependence in MRT59 patients. This is the first study to investigate those two variables in patients with *IMPA1*-associated intellectual disability. Regarding the ICD-10 (classification of severity based on IQs), our results (IQs ranged from 40 to 44) revealed moderated intellectual disability [14] in all *IMPA1* patients. On the other hand, based on the DSM-5 criteria (classification of severity regarding the functionality) [15], MRT59 participants showed moderate to severe intellectual disability (FIM ranged from 29 to 101). Looking at only one dimension, either IQ or daily skills, is not enough for diagnosing intellectual disability. A combination of the ICD-10 and DSM-5 criteria is important in promoting an integrated view of the individual.

In line with this, the combined results pointed to moderate to severe intellectual disability in MRT59 individuals. Participants’ IQs were low according to the WASI classification and the lowest indexes of FIM were found in the communication, social cognition and self-care domains. Our results corroborate those reported by Figueiredo et al. (2016) [5]. Additionally, the IQs were positively correlated with communication abilities and social cognition.

MRT59 participants showed low performance in verbal comprehension (vocabulary and similarities) and nonverbal fluid ability subtests (block design and matrix reasoning). They showed reduced vocabulary and impairments in the ability of analysis and synthesis; executive functions; integration of knowledge; visual and spatial perception; processing speed and cognitive flexibility. A recent study using electroencephalogram (EEG) reported alpha-band abnormalities in MRT59 individuals, which could be related to the cognitive deficits observed in our patients [6].

Low performance on the similarities subtest has been reported in patients with bipolar disorder [16]. Despite the higher frequency of psychiatric symptoms in MRT59 patients, bipolar disorder has been associated with changes in *IMPA2* and not in *IMPA1* [17].

Intellectual disability associated with *IMPA1* (MRT59) was initially described in a large consanguineous family from the hinterlands of Northeast Brazil. Consanguinity is fairly common in these backlands, and high consanguinity rates may increase the probability of autosomal recessive diseases, as has been previously reported in cerebellar ataxias [18]. Additionally, high consanguinity rates increase the yield of genetic prospection for new disease-associated genes and variants. Consequently, other autosomal recessive intellectual disability genes have been also described in this same population, such as *MED25* [19].

The SPOAN syndrome (an acronym for spastic paraplegia, optic atrophy, and neuropathy syndrome) is a new autosomal recessive genetic condition that was discovered in an isolated region of the State of Rio Grande do Norte in Northeast Brazil, in a population that was identified by the IBGE (Brazilian Institute of Geography and Statistics) as belonging to the Brazilian communities with the highest rates of “deficiencies”, a term used to describe diseases, malformations, and handicaps in general.

This motivated our group to conduct research on consanguinity levels in five of its municipal areas, which we did by conducting direct interviews with their residents. Data on 7639 couples (about 40% of the total population of the study areas) were acquired. The study found unusually high rates of consanguineous marriages ranging from roughly 9% to 32%, indicating the presence of a clear link between genetic illnesses like SPOAN syndrome, genetic drift, and inbreeding levels [20].

This led to the development of epidemiological strategies to collect inbreeding data with the collaboration of health systems available in the region, for the prospecting of genetic disorders. This effort resulted in the identification of at least two more autosomal recessive conditions in this region, namely *IMPA1*-associated intellectual disability (MRT59) and *MED25*-associated syndromic intellectual disability, a condition with typical facial dysmorphisms characterized by tall forehead, prognathism, prominent chin and very large and overhanging nose tip [19].

The social background of these populations with low education levels underlines the need for specific neuropsychological evaluation tools for characterizing the degree of intellectual disability, as well as the most involved cognitive domains in these patients. Therefore, the present study may serve as the basis for the conduction of further cognitive assessment research in this specific inbred population of Northeast Brazil.

A homozygous loss-of-function variant in *IMPA1* was initially described in nine individuals with severe intellectual deficiency belonging to a large consanguineous family of the hinterlands of Northeast Brazil. *IMPA1* encodes inositol monophosphatase, an enzyme of the final step of dephosphorylation of polyphosphate myo-inositol. However, changes in plasma and urine myoinositol or phosphoinositol were not detected in affected patients and brain magnetic resonance imaging spectroscopy did not show alterations in parenchymal inositol concentrations, suggesting that extracellular fluids were not affected by IMPA1 enzyme deficiency. Intracellular inositol changes were proposed as a possible disease mechanism, but these were difficult to assess at this moment [7].

Some studies evaluated the effects of *IMPA1* pathogenic variants in mice [3]. Cryns et al. (2008) [3] revealed high embryonic lethality in homozygote Impa1−/− mice between days 9.5 and 10.5 post coitum. Interestingly, inositol supplementation to pregnant mothers rescued these embryos. However, behavior analysis of rescued mice showed hyperactivity despite inositol supplementation, as well as altered circadian cycle control. Additionally, Berry et al. (2003) [21] have shown that homozygous deletion of the sodium myo-inositol cotransporter-1 (SMIT) in mice, the product of which is responsible for importing inositol into cells, resulted in mortality shortly after birth. Ohnishi et al. (2014) [22] examined an ethyl-nitrosourea mutant library for Impa1/mutations and discovered a Thr95Lys missense mutation that caused perinatal mortality in mice but could be restored by inositol administration. Homozygotes had hyperlocomotive behavior as well as extended circadian intervals, similar to what has been found in *IMPA1* knockout mice. According to Andreassi et al., (2010) [23], *IMPA1* messenger RNA is the most abundant transcript in rat sympathetic neuron axons, and specific silencing of *IMPA1* causes axon degeneration.

These findings emphasize the role of myo-inositol in mouse embryonic growth and survival, and they imply that inositol deficiency, whether caused by insufficient synthesis, recycling, or transport, is deleterious to normal development. The rescue of mortality in the Impa−/− mice by inositol supplementation, on the other hand, suggests that increased food supplementation can compensate for the deficit in myo-inositol recycling and synthesis in the developing embryo. The effects of inositol supplementation in adult-affected patients remain to be studied.

Following these findings, a study with induced Pluripotential Stem Cells (iPSCs) was conducted by Figueiredo et al. (2021) [7] to further investigate the molecular and cellular mechanisms involved in intellectual disability associated with *IMPA1* loss-of-function (MRT59). This study showed that IMPA1 enzyme deficiency did not affect iPSCs, glial progenitor cells (GPCs) and astrocyte differentiation. However, IMPA1 deficiency had a significant effect on neuronal progenitor cells (NPCs) leading to cell cycle arrest, neuronal apoptosis and poor neurogenic capability in these cells. As passages increased, these cells stopped proliferating and shifted to a gliogenic progenitor phenotype. Interestingly, transcriptome analysis of 2-week-old neurons derived from *IMPA1*-mutated patients revealed increased expression of glial markers (S100B, S100A2, PAX6, GFAP, ALDH1L1, and CD40). When these NPCs were cocultured with primary astrocytes neurogenesis did not improve, suggesting the mechanism to be cell-specific and a direct result of the *IMPA1* mutation on neuronal progenitors. There was a significant improvement in neurogenesis after inositol supplementation when provided to iPSCs before complete neuronal differentiation, but not when it was started from NPCs [7]. These results are in line with animal model studies where *IMPA1*−/− mice were rescued in the embryonic phase with maternal supplementation of inositol during the gestational period. Those combined findings emphasize the relevance of inositol in early embryonic development and imply that inositol deficit, whether caused by insufficient synthesis, recycling, or transport, is deleterious to proper neuronal development.

Additionally, transcriptome studies revealed that neuronal progenitor cells and neurons generated from patients with intellectual deficiencies have widespread gene expression dysregulation impacting neurogenesis-related pathways such as Wnt and GPCR signaling [24,25]. Furthermore, NPCs derived from patients with mutant *IMPA1* showed significantly increased expression of many gliogenesis-related genes, as well as considerable overexpression of Hes3 and Hes5, which have been demonstrated to prevent neuronal differentiation and preserve neural stem cells in the embryonic brain [26,27,28]. Those findings also point to a shift from neuronal to glial differentiation in NPCs of IMPA-deficient subjects.

Upregulation of genes involved in oxidative phosphorylation and other mitochondria-related genes has been detected in the frontal cortex of *IMPA1* and *SMIT1* knockout mice, suggesting an interplay between inositol synthesis and recycling and mitochondrial function [4]. However, transcriptome analysis of NPCs derived from *IMPA1* patients did not show substantial differences in mitochondrial gene expression. Despite this fact, mitochondrial dysfunction cannot be ruled out as part of the pathophysiology of MRT59, as apoptotic NPCs derived from these patients show increased expression of *ITPR3* and *ITPR2*, genes involved in excessive calcium accumulation in mitochondria leading to reactive oxygen species accumulation, senescence and apoptosis. Thus, a role for mitochondrial dysfunction in NPC apoptosis and decreased neurogenic potential in IMPA1 deficiency is a possibility [7].

The general interest in IMPA1 function has steadily risen over the years since it was discovered that this enzyme is suppressed by lithium, a potent mood stabilizer used to treat bipolar disorder, a psychiatric ailment with a high global prevalence. Several research studies have attempted to comprehend lithium’s mode of action and its relationship to IMPA1 activity.

Only *IMPA1* and *IMPA2* have been shown to encode proteins with inositol monophosphatase activity in humans. Nonetheless, lithium inhibited IMPA2 only at high doses and it had significantly lesser inositol monophosphatase activity than IMPA1 [29]. *IMPA1* and *IMPA2* exhibit varied patterns of expression in various tissues in mice. *IMPA1* dominates expression in the brain, for example. Furthermore, *IMPA2* knockout mice have no discernible phenotype. In the search for putative compensating mechanisms, there was no indication of Impa2 overexpression in Impa1/mice [3]. According to research on the Caenorhabditis elegans gene, ttx-7, which encodes an inositol monophosphatase, IMPA1 plays an important role in maintaining neuronal polarity in the adult nervous system [30,31]. Ttx-7 mutations impair sensory behavior and the location of both pre- and post-synaptic proteins in RIA neurons and key interneurons. Ttx-7 mutants’ behavioral and localization abnormalities were repaired by adult ttx-7 expression, forced expression of human IMPase proteins, or inositol supplementation [30,31,32]. Tanizawa et al. (2006) [31] demonstrated that synaptic localization abnormalities in ttx-7 mutants occurred exclusively in RIA neurons. As a result, we may assume that inhibiting IMPA1 in humans may influence a specific set of neurons and/or a specific metabolic phosphatidylinositol pathway.

The absence of a decreased inositol peak in one MRT59 patient that underwent brain magnetic resonance imaging spectroscopy does not rule out the potential of inositol depletion as a mechanism of intellectual disability. Previous research has revealed that the brain has several pools of inositol [3,33]. Lack of IMPA1 activity leading to inositol depletion would be more likely in cells with a very active phosphatidylinositol cycle, resulting in inositol depletion and permanent brain damage [3]. 

Several intellectual deficiency genes are linked to the release of neurotransmitters via exocytosis [34]. Phosphoinositides, which are formed by the combinational phosphorylation of phosphatidylinositol, have been demonstrated to play key roles in the Ca^2+^-dependent mobilization of secretory vesicles to the plasma membrane [35,36]. Regulated secretion in neurons and neuroendocrine cells involves a calcium-dependent fusion of transmitter-containing vesicles with the plasma membrane. Furthermore, phosphatidylinositol 3,5-bisphosphate deficiency promotes neuroexocytosis and neuronal degeneration, a process connected to Charcot–Marie–Tooth disease and amyotrophic lateral sclerosis [37].

Our group conducted a previous study to evaluate the neurophysiologic mechanisms that mediate the *IMPA1*-associated intellectual disability phenotype. There were no theories of electrophysiological biomarkers for MRT59 since there had been no prior human or animal neurophysiology research on the *IMPA1* mutation. As a result, the most similar pharmacological model of IMPA1 inhibition was used: lithium. Previous mouse research revealed that lithium works by inhibiting IMPA1 and decreasing Smit1 mRNA expression [38]. In illustration, lithium inhibition of inositol monophosphatase increased IP1 (inositol phosphate-1) concentrations in cell cultures, which influenced the pace of phosphoinositide production [39]. It was expected that IMPA1 deficiency would cause a similar disruption of the inositol metabolic cycle as lithium administration. Lithium has been shown in human EEG research to improve early sensory potentials, increase low-frequency activity in resting state EEG, and increase event-related beta oscillations [40,41]. Thus, homozygous carriers of a loss of function *IMPA1* mutation were expected to behave similarly to healthy controls under chronic lithium treatment and have more low-frequency activity (i.e., delta, theta, and alpha oscillations) than carriers of the wild-type version of *IMPA1*.

However, that study has found evidence of reduced frontal theta oscillations in association with intellectual disability in MRT59. Theta oscillations have been associated with cognitive control functions such as error monitoring [42] and working memory [43,44], including the modulation of local gamma activity [45] and mediating cortical interactions with the hippocampus [46]. These findings were likely to be associated with poor working memory and cognitive control, but the lack of a neuropsychological assessment at that point could not confirm this hypothesis. The present study of neuropsychological assessment showing specific impairments in executive functions, processing speed and cognitive flexibility finally corroborates the association of these neurophysiological findings with the profile of cognitive deficits in MRT59.

All MRT59 patients had behavioral abnormalities, such as agitation and social withdrawal, but none of them fulfilled the DSM-V criteria for bipolar disorder or schizophrenia.

Some limitations of our study need to be addressed. First, we did not evaluate psychiatric disorders in this population. Second, neuroimaging evaluation of all MRT59 patients would have been informative to investigate the relation between brain abnormalities, cognitive impairments and functional dependence. Neuroimaging was carried out in only one affected patient and did not show significant abnormalities.

The recognition of the genetic basis and pathological mechanisms of this disease created the means for adequate genetic counseling. Studies will be needed to examine whether early supplementation with inositol could bring benefits to MRT59 patients.

## 5. Conclusions

Our findings revealed that patients MRT59 patients are mostly affected by verbal and executive cognitive functions. If we used only the psychometric criteria of ICD-10, MRT59 patients would be classified as having moderate to severe intellectual disability, but when we also evaluate them with a dependency assessment tool, we understand that they have moderate to severe disability according to the DSM 5 criteria due to relevant dependence on their caregivers. MRT59 patients should be followed up by health professionals in an interdisciplinary way to understand their cognitive disabilities and functional needs properly.

## Figures and Tables

**Table 1:** Description of sample (*n* = 18) (MRT59 vs. carriers vs. non-carriers).

Characteristics	MRT59 (*n* = 9)	Carriers (*n* = 5)	Non-Carriers (*n* = 4)	*p*
Age mean (Range)	53 (43–64)	69 (54–89)	55 (37–75)	0.99
Education (*n*)				0.78
No education or semi-literate	9	2	2	No education or semi-literate
Elementary school		1	0	
High school		0	1	
College		2	1	

**Table 2:** QI scaled scores of MRT59, carrier and non-carrier participants.

	MRT59 (*n* = 9)	Carriers (*n* = 5)	Non-Carriers (*n* = 4)
VIQ mean (SD)	47.11 (2.80)	77.00 (14.81)	87.50 (19.05)
PIQ mean (SD)	46.00 (1.58)	85.40 (10.99)	87.50 (15.99)
FISQ-4 mean (SD)	41.33 (1.80)	75.20 (12.85)	86.25 (18.19)
FISQ-2 mean (SD)	43.33 (5.07)	79.20 (13.21)	91.75 (12.55)

## Data Availability

All data were included in the manuscript or supplementary material.

## Supplementary Materials

The following supporting information can be downloaded at: https://www.mdpi.com/article/10.3390/brainsci13071048/s1, Table S1: sociodemographic data and WASI performance of individual participants; Table S2: functional dependence data in MRT59 participants (*n* = 9).
