# Supplementary material for: Neuropsychological Characterization of Autosomal Recessive Intellectual Developmental Disorder 59 Associated with IMPA1 (MRT59)
Source: Brain Sci. 2023 Jul 10;13(7):1048. doi: 10.3390/brainsci13071048 (PMC10377093; doi:10.3390/brainsci13071048)
Supplement: Supplementary file 1 [file brainsci-13-01048-s001.zip › brainsci-2490704-supplementary.pdf]

Table S1. Sociodemographic data and WASI performance of individual participants

Participants	Group	Age	Education	VC	BD	SM	RM	VQI	PIQ	FSQI-4
**1**	MRT59	54:2	SL	0/1	0/1	0/1	0/1	45	45	40
**2**	MRT59	48:0	SL	17/3	0/1	3/1	1/1	52	47	44
**3**	MRT59	50:5	SL	11/2	0/1	2/1	3/2	48	49	43
**4**	MRT59	64:1	SL	3/1	0/1	4/2	0/1	48	45	41
**5**	MRT59	43:8	SL	0/1	0/1	0/1	0/1	45	45	40
**6**	MRT59	60:5	SL	0/1	0/1	0/1	0/1	45	45	40
**7**	MRT59	56:10	SL	8/1	0/1	1/1	0/1	45	45	40
**8**	MRT59	53:2	SL	16/3	0/1	2/1	2/2	51	48	44
**9**	MRT59	51:0	SL	2/1	0/1	2/1	0/1	45	45	40
**10**	carrier	54:4	C	49/10	28/9	27/8	20/8	93	93	91
**11**	carrier	63:0	ES	23/4	9/5	9/3	7/4	64	70	63
**12**	carrier	89:11	SL	29/6	4/6	11/5	9/10	76	90	79
**13**	carrier	84:9	SL	24/5	2/4	2/2	2/5	61	69	61
**14**	carrier	55:4	C	41/8	10/6	28/9	13/6	91	78	82
**15**	non-carrier	46:9	HS	58/8	29/6	36/9	26/6	107	99	104
**16**	non-carrier	75:10	SL	27/6	2/4	2/1	9/8	62	77	66
**17**	non-carrier	37:00	C	46/9	49/11	34/9	25/10	95	102	99
**18**	non-carrier	65:03	SL	45/9	5/5	17/6	8/5	86	72	76

VC: Vocabulary; BS: Block Design; SM: Similarities; RM: Matrix Reasoning; S.L: semi-literate or no education; ES: elementary school; HS: high school; C: college. VC, BS, SM e RM were expressed using raw and scaled scores (r/s), respectively.

Table S2. Functional dependence data in MRT59 participants (n=9)

Patient	Self-Care						Sphincter Control					Locomotion		Comunication		Social cognition			Total	Mean
	E	G	B	UBD	LBD	T	BM	BoM	B/C/Wh	T	B/S	W/Wh	S	C	Ex	SI	SP	M		
1	2	1	1	2	2	1	7	7	7	7	7	7	4	2	2	2	2	2	65	3.61
2	2	1	2	6	6	1	7	7	7	7	7	7	4	4	4	4	4	4	84	4.66
3	4	4	4	3	3	3	7	7	7	7	7	7	7	4	4	5	2	3	88	4.88
4	2	2	2	6	6	1	6	6	6	6	6	7	6	2	2	3	3	3	75	4.16
5	2	1	1	2	2	1	1	2	6	6	6	6	2	1	2	1	1	2	45	2.50
6	2	1	1	2	1	1	1	1	2	1	1	3	3	2	2	2	1	2	29	1.61
7	2	6	6	6	6	6	7	7	7	7	7	7	5	3	3	5	2	3	95	5.27
8	3	4	6	6	6	6	7	7	7	7	7	7	5	4	4	5	5	5	101	5.61

Self- care: F – eating; SC - grooming; B – bathing; UBD – upper body dressing; LBD – lower body dressing; T- toileting; Sphincter control: BM - Bladder management; BoM – Bowel management; Mobility: B/C/Wh – bed/chair/wheelchair; T- toilet; B/S- bath, shower; Locomotion: W – walking, Wh – Wheelchair; S - stairs Communication: C – comprehension; E – expression; Social cognition: SI – social interaction; SP – solving problem; M – Memory.
